# Relationship between Children’s Intelligence and Their Emotional/Behavioral Problems and Social Competence: Gender Differences in First Graders

**DOI:** 10.2188/jea.JE20090146

**Published:** 2010-03-05

**Authors:** Lian Tong, Ryoji Shinohara, Yuka Sugisawa, Emiko Tanaka, Taeko Watanabe, Yoko Onda, Yuri Kawashima, Yuko Yato, Noriko Yamakawa, Tatsuya Koeda, Hiraku Ishida, Shinako Terakawa, Ayumi Seki, Tokie Anme, Japan Children’s Study Group

**Affiliations:** 1 Research Institute of Science and Technology for Society, Japan Science and Technology Agency, Tokyo, Japan; 2 Graduate School of Comprehensive Human Sciences, University of Tsukuba, Tsukuba, Ibaraki, Japan; 3 Ritsumeikan University, Kyoto, Japan; 4 Clinical Research Institute Mie-Chuo Medical Center National Hospital Organization, Tsu, Japan; 5 Department of Regional Education, Faculty of Regional Sciences, Tottori University, Tottori, Japan; 6 Research Fellow, Japan Society for the Promotion of Science, Tokyo, Japan; 7 Junior College, Gifu Shotoku Gakuen University, Gifu, Japan

**Keywords:** children, intelligence, emotional and behavioral problems, social competence, parenting

## Abstract

**Background:**

The present study examines gender differences in the correlations between intelligence and developmental problems as well as social competence in first graders.

**Methods:**

Ninety parent-child dyads participated in this study. The children comprised 7-year-olds recruited from the first grade of an elementary school. All the children were administered the Wechsler Intelligence Scale for Children–Third Edition (WISC-III), Parent-child Interaction Rating Scale (IRS), and the parent report version of Strength and Difficulties Questionnaire (SDQ).

**Results:**

The findings clarified that the processing speed of boys significantly correlated with their peer relationship. On the other hand, the emotional symptoms exhibited by girls had a more common association with their intellectual abilities. The correlations between parenting and intellectual abilities differed in boys and girls.

**Conclusions:**

Children’s gender should be taken into account when assessing the diversity in their intellectual abilities and developmental problems. Moreover, parenting also influences the development of children in various ways.

## INTRODUCTION

Though many studies have found no or negligible gender differences in the overall cognitive ability,^1^^,^^2^ when looking at more specific abilities—where mean differences are found more often—the extent of gender differences has not yet been estimated. It is often reported that girls score higher than boys in terms of verbal skills; however, boys usually outperform girls when it comes to numerical skills.^3^ Many studies have examined the gender differences in the relationship between children’s intelligence and academic performance.^4^^,^^5^ Children who scored higher on cognitive assessments after controlling for family demographics, and parenting showed a high level of social competence. However, the specific differences in gender profiles were not clarified. Furthermore, the gender differences in the correction between children’s intellectual abilities and developmental problems have not yet been extensively investigated.

Emotional and behavioral disturbances are widely prevalent among school-aged children. The morbidity of developmental problems in children shows obvious gender differences in specific aspects. Studies carried out in various countries confirm that 4%–9% of elementary school boys suffer from emotional or behavioral problems.^6^^,^^7^ According to a survey conducted in an Australian early primary school, 5.3% of the schoolboys had scores within the abnormal clinical range on four of the five subscales of the Strength and Difficulties Questionnaire (SDQ).^8^

When children enter school, they become increasingly involved in extrafamilial relationships. It is also a time when external influences take on greater importance in influencing what children do and the choices they make.^9^ During this time, genetic and environmental influences that contribute to the dissimilarity of individuals growing up together become increasingly important, probably because of the parenting factor or home environment.^10^^,^^11^ Few studies have addressed these influences with regard to children’s individual characteristics, including cognitive ability and gender differences.^12^

Although the time spent by parents with school-aged children has decreased, parents continue to have a strong influence on children. The research conducted by Wigfield and Eccles^13^ demonstrated that children’s self-concepts of ability are primarily influenced by their parents’ beliefs, expectations, attitudes, and behavior. Social gender roles were traditionally defined by taking into consideration the rearing practices and attitudes of parents in daily parent-child interactions. Therefore, in addition to children’s individual factors, parents’ rearing practices and gender stereotypes are taken into consideration in this study. This study aims to examine the gender differences in the correlations between children’s intellectual abilities and their social competence and emotional/behavioral problems. We also aim to demonstrate the relationship between parenting and children’s intellectual development in first graders.

## METHODS

### Participants

Ninety first graders were randomly selected from an elementary school located in a mid-sized city in Japan. Their parents were also recruited in this study. Before the commencement of the research, all the participants were required to read and sign an informed consent form (Parents make signatures for their children in this study due to the age of children). They were made aware that they could withdraw at any time. Both the contents and procedure of this research were approved by the ethics committee of the Japan Science and Technology Agency (JST). The children’s primary caregivers (mostly mothers) were also recruited for the study.

### Questionnaires evaluation and coding

The Wechsler Intelligence Scale for Children–Third Edition (WISC-III)^14^ was used to evaluate the children’s intelligence level. The test comprises 13 subtests. These subtests then generate a Full Scale IQ (FSIQ), Verbal IQ (VIQ), and Performance IQ (PIQ) scores, as well as four composite scores (indices): Verbal Comprehension Index (VCI), Perceptual Organizational Index (POI), Processing Speed Index (PSI), and Working Memory Index (WMI). The VCI subtests cover comprehension of vocabulary, similarities, and information; the POI subtests include block design, picture arrangement, object assembly, matrix reasoning, and picture completion; the WMI subtests contain digit span and arithmetic; and the PSI subtests include coding and symbol search.

The parent report version of the SDQ was used to evaluate the children’s emotional and behavioral development. The questionnaire comprises 25 items, organized into the following five subscales: hyperactivity, emotional problems, conduct problems, peer relationships, and prosocial behavior. Each item is accompanied by the response options “1 = Not true,” “2 = Somewhat true,” and “3 = Certainly true.” The total score for each subscale (0–10) is obtained by adding the scores obtained for the five items that compose the subscale. Finally, the scores for the subscales of hyperactivity, emotional problems, conduct problems, and peer relationships are added to obtain a total score ranging from 0 to 40 (total SDQ score). A higher score indicates a higher risk of the child developing difficulties in the areas represented by the subscales. The score for the prosocial subscale is not incorporated in the total score, since the absence of prosocial behaviors is conceptually different from the presence of psychological difficulties.^15^ Converse to the other subscales, a higher score for the prosocial behavior subscale indicates a lower risk of developing prosocial behavioral problems.

### Laboratory observation and coding

The children’s social competence and the caregiver’s parenting were evaluated using the Interaction Rating Scale (IRS), which was modified on the basis of the Nursing Child Assessment Satellite Training (NCAST)–Teaching Scale^16^ and which is applicable to children aged 0 to 8 years. The IRS items are is organized into the following five subscales pertaining to the child’s competence: (i) autonomy, (ii) responsiveness, (iii) empathy, (iv) motor self-regulation, and (v) emotional self-regulation. In addition, it contains the following five subscales pertaining to the caregiver’s competence: (i) sensitivity toward the child, (ii) responsiveness to the child, (iii) intrusiveness to the child, (iv) fostering of the child’s socioemotional growth, and (v) fostering of the child’s cognitive growth. Internal consistency in each category, as measured by Cronbach’s alpha, ranged from .43 to .88, and the total internal consistency was excellent (.85–.91). Additionally, the IRS showed highly significant correlations with the NCAST teaching scales (items relating to the child: *r* = .70, items relating to the caregiver: *r* = .98, total: *r* = .89).^17^

In this study, the IRS was evaluated as follows. Five-minute video recordings of the child-caregiver interaction (the child and caregiver playing with blocks and putting them in a box) for each dyad was carried out. The caregiver-child interactions were videotaped in a controlled laboratory environment—a room with five video cameras, one camera placed in each of the four corners of the room and one in the centre of the ceiling. The dyads were escorted to the room (4 × 4 m), which was furnished with a small table and a small chair meant for children. The caregiver introduced himself/herself to the child and interacted with the child in a natural manner, just as he/she would on a regular day.

### Assessing the assessors

Two members of the research team coded the observed behaviors. A third child professional, who had no contact with the participants, also scored the behaviors. The behaviors of the children and caregivers during the caregiver-child interactions were coded as follows. If the child displayed the behavior described in the item, a score of 1 was given; conversely, if the child failed to display the behavior described in the item, a score of 0 was given. A higher score indicated a higher level of development in the child. The same method of coding was used to evaluate the parenting practice.

### Analysis

The Statistical Analysis System (version 9.1) was used for the data analysis. The correlation analysis was conducted to examine the relations between the factors. A Pearson correlation coefficient was used to evaluate the correlation at the significance level of *P* < 0.05.

## RESULTS

Of the 90 children who participated in the study, 49 were boys (53.9%) and 41 were girls (46.1%). At the time of the study, only 9 participants (10.0%) were the only child of their parents; 58 (64.5%) had one sibling, 20 (22.2%) had two siblings, and 2 (2.2%) had three siblings. Furthermore, of all the participating parents, 61 (67.8%) mothers and 54 (60.0%) fathers fell in the age group of 35–45 years. Of the 90 children, 47 (52.2%) lived with both parents; 3 (3.3%) lived with a single mother; 40 (44.5%) lived in an extended family, from which 34 (37.8%) lived with their parents and grandparents, 5 (5.6%) lived with a single mother and grandparents, and 1 (1.1%) lived with a single father and grandparents. Other relevant demographic information on the children and families is presented in Table 1.

**Table 1.** Participant Demographics

Items	No	Rate (%)
Age		
7 years	90	100.0
Gender		
Boys	49	53.9
Girls	41	46.1
Siblings		
0	9	10.0
1	58	64.5
2	20	22.2
3	2	2.2
NA	1	1.1
Mother’s age		
25–35	20	22.2
35–45	61	67.8
45–55	6	6.7
NA	3	3.3
Father’s age		
25–35	14	15.6
35–45	54	60.0
45–55	12	13.3
NA	10	11.1
Family type		
Nuclear family		
Parents	47	52.2
Mother only	3	3.3
Extend family		
Parents, grandparents	34	37.8
Mother, grandparents	5	5.6
Father, grandparents	1	1.1
Family income		
<2 million JPY	2	2.2
2–4 million JPY	17	18.9
4–6 million JPY	33	36.7
6–8 million JPY	20	22.2
8–10 million JPY	9	10.0
≧10 million JPY	5	5.6
NA	4	4.4

Total	90	100.0

JPY denotes Japanese yen.

The difference was apparent in the correlations between the children’s intellectual development and their developmental problems. The results indicated that the boys’ processing speed (PSI) negatively correlated with their developmental problems in peer relationships (*r* = −0.45, *P* < 0.01); in other words, boys with a high processing speed would have less problems in peer relationships than those with low processing speeds (Table 2). In comparison, the girls’ intelligence levels were more correlated with their emotional and behavioral problems (Table 3). The girls’ FIQ scores and their intelligence in perceptual organization (*r* = −0.34, *P* < 0.05) had a general negative correlation with their total score of emotional and behavioral problems (*r* = −0.38, *P* < 0.05). It is worth noting that the girls’ intellectual development significantly correlated with their emotional symptoms. Their general intelligence level (FIQ) negatively correlated with their emotional symptoms (*r* = −0.42, *P* < 0.05); meanwhile, their emotional symptoms also correlated with their PIQ (*r* = −0.36, *P* < 0.05) and POI (*r* = −0.41, *P* < 0.05). This indicates that girls with a higher intelligence level, especially in terms of performance and perceptual organization, will exhibit less emotional problems. Otherwise, we did not find any correlation between intelligence level and other aspects of behavioral problems.

**Table 2.** Correlation between boys’ intelligence levels and emotional/behavioral problems

	Peer relationship
Processing Speed (PSI)	−0.45^a^

^a^*P* < 0.01.

**Table 3.** Correlation between girls’ intelligence levels and emotional/behavioral problems

	SDQ’s total score	Emotion symptoms
Full Scale score (FIQ)	−0.38^a^	−0.42^a^
Performance IQ (PIQ)	−0.30	−0.36^a^
Perceptual Organizational (POI)	−0.34^a^	−0.41^a^

^a^*P* < 0.05.

The correlation analysis results showed that parenting is highly correlated with the children’s intellectual development in the case of boys (Table 4). Specifically, the arithmetic skills of the boys positively correlated with the parents’ respect for their autonomy (*r* = 0.35, *P* < 0.05). Their scoring in the block design subtest positively correlated with the parent’s sensitivity toward them (*r* = 0.35, *P* < 0.05). Meanwhile, we found that the girls’ intellectual levels had a general correlation with both parenting and their individual social competence development. The results show that the girls’ VIQ (*r* = 0.37, *P* < 0.05), VCI (*r* = 0.36, *P* < 0.05), and scoring in vocabulary (*r* = 0.50, *P* < 0.05) were positively correlated with their autonomy development (Table 5). Additionally, the girls’ intelligence levels were pervasively correlated with their parent’s sensitivity toward them. In particular, the girls’ scoring in VIQ (*r* = 0.53, *P* < 0.01) and factor index scoring in VCI (*r* = 0.47, *P* < 0.01), WMI (*r* = 0.41, *P* < 0.05), and PSI (*r* = 0.41, *P* < 0.05) are positively correlated with their parents’ sensitivity toward them. Their factor index scoring of similarities showed a high correlation with the parents’ sensitivity (*r* = 0.64, *P* < 0.05) and responsiveness to the children (*r* = 0.39, *P* < 0.05). Besides, parents’ responsiveness to children positively correlated with the girls’ VIQ (*r* = 0.38, *P* < 0.05). We also found that children whose parents aimed to foster their cognitive growth performed well in the comprehension of similarities subtest (*r* = 0.37, *P* < 0.05).

**Table 4.** Correlation between boys’ intelligence levels and parenting

	Parents’ items	

	Sensitivity	Respect for child’s autonomy
Arithmetic	−0.12	0.35^a^
Block Design	0.35^a^	0.14

^a^*P* < 0.05.

**Table 5.** Correlation between girls’ intelligence levels and girls’ social competence/parenting

	Girls’ item	Parents’ items		
	
	Autonomy	Sensitivity	Responsiveness	Fostering of cognitive growth
Full Scale score (FIQ)	0.31	0.46^b^	0.26	−0.17
Verbal (VIQ)	0.37^a^	0.53^a^	0.38^a^	0.04
Verbal Comprehension (VCI)	0.36^a^	0.47^b^	0.36^a^	0.06
Working Memory (WMI)	0.30	0.41^a^	0.20	−0.21
Processing Speed (PSI)	0.23	0.41^a^	0.08	−0.13
Similarities	0.24	0.64^a^	0.39^a^	0.37^a^
Arithmetic	0.22	0.49^b^	0.27	−0.10
Block Design	0.22	0.43^a^	0.30	−0.16
Vocabulary	0.50^b^	0.31	0.23	0.16
Comprehension	0.17	0.40^a^	0.35	0.02
Symbol search	0.29	0.51^b^	0.20	−0.12

^a^*P* < 0.05, ^b^*P* < 0.01.

## DISCUSSION

The obvious differences among the sample of first graders were observed in the correlations between their intelligence levels, and their developmental problems and social competence. The results indicated that the boys’ processing speeds highly correlated with their peer relationship. The scoring of PSI requires visual perception and organization, visual scanning, and the ability to use hands/eyes together in an efficient manner. It probably affects boys’ performance in peer related activities. Attention also is required to complete the time limited coding and symbol search during PSI subtests. As the previous study indicated that girls are much quicker than boys at timed tasks, although kindergarten and younger children process tasks at similar speeds, the difference becomes pronounced in elementary school.^18^ Furthermore, boys tend to be characterized by mischievousness, like engaging in outside activities and being more engaged in sports. As school-aged children, they spend a considerable amount of time with their peers. Children interact more with peers in the classroom, while playing sports, and during after-school programs. In such settings, children prefer playing with other children of the same gender and tend to stereotype members of the opposite gender. The processing speed of boys affects their sports skills, self-esteem building, and peer group acceptance, thus eventually affecting their peer relationship.

On the other hand, girls’ holistic intellectual level is negatively correlated with their emotional and behavioral problems; in particular, girls’ emotional symptoms are generally correlated with their PIQ and the POI scoring. Generally PIQ was considered to be related with children’s behavior problems. The previous study also indicated that the PIQ predict emotion recognition ability in children with autistic spectrum disorders children.^19^ The finding in the present study offer more evidence on it. The POI is a measure of non-verbal and in-the-moment reasoning. It assesses the ability to examine a problem, draw upon visual-motor and visual-spatial skills, organize thoughts, create solutions, and then test them. It is a set of mental activities that includes thinking, knowing, and remembering.^20^ The associations obtained in the present study predict the potential relationship between girls’ perceptual organization and their emotional problems.

In addition to the relationships with developmental problems, we aimed to clarify the relationship between intelligence and social competence among first graders. Though no clear relationships were found in the boys’ profile, the girls’ ability in vocabulary and their verbal comprehension were found to be highly associated with the development of their autonomy. The popular conception is that girls develop all communication skills earlier than boys. Vocabulary and effective expressions are skills necessary for children to express their autonomy. On the other hand, children whose autonomy is well established frequently communicate with parents or peers, possibly promoting their capability of expressing verbally.

In this study, parenting practices, especially the parents’ sensitivity toward children, were found to be significantly correlated with the children’s intelligence in both boys and girls. In particular, the girls’ intelligence highly correlated with the parent’s sensitivity toward them. The indexes of VCI, WMI and PSI significantly correlated with parent’s sensitivity. The child’s gender often predicted his/her involvement, responsiveness, and communicativeness during the interactional task; maternal sensitivity mediated some of these relations.^21^ Gender differences in the correlations might be explained in part by the differential treatment of boys and girls by their mothers. In Japan, girls are frequently trained to conform to traditional gender roles, which include exhibiting the traits of calmness, politeness, and attentiveness to the needs of others.^22^ In the present study, most of the caregivers were mothers, who are generally considered to be more sensitive toward girls than boys.

The findings also indicate that the parents’ responsiveness was related to the girls’ verbal IQ and the scoring in the similarities subtest. The fostering of the girls’ cognition development by the parents partially benefited their intellectual growth. It indicated that the approach of parents positively interacting with girls and consciously teaching them during their daily interactions were good indicators of their intellectual development.

Compared to girls, diverse correlations were found in boys. The parents’ respect for the boys’ autonomy was positively associated with their arithmetic skills. Respect for autonomy implies that one should be free from coercion in deciding to act and that others are obligated to protect confidentiality, respect privacy, and tell the truth. The existing studies indicate that mothers’ high directiveness, external control, and concentrate on the ongoing activity by using many imperatives, made the children regulation of toddlers’ behavior, such as encouraging toddlers to be attentive and more successful in solving the puzzle tasks meant for older children; however, more respect for their autonomy would facilitate their development.^23^^,^^24^ Additionally, boys tend to perform better in math; this is possibly another reason explaining parents’ high respect for the boys’ autonomy, since they believe that boys will do well in that field.

Not much is known about the gender differences in the correlations between parenting and children’s intelligence; however, similar gender differences have been indicated in existing studies. For instance, one study highlights the gender differences in the correlations among parent and child anxiety sensitivity. It indicated that 42% of the effect of parent anxiety sensitivity on laboratory pain intensity was explained by girls’ anxiety sensitivity. In boys, there was no clear association between them.^25^ A similar trend was found in this study. In sum, the findings obtained in the present study suggested that gender differences should be taken into account for investigating the differences in children’s emotional and behavioral problems and the correlations of these factors with their intelligence. Meanwhile, different parenting approaches should be adopted toward boys and girls depending on the diversity of their intellectual development.

It is worth noting the strengths and limitations of the present study. The findings clarified in detail the gender differences in the development of first graders. It offers new evidences on the association between children’s intelligence and developmental problems as well as the social competence in both gender profiles. It also identified the key associations between parenting practices and children’s intelligence. However, this study has certain shortcomings. The investigations were carried out in the same year; therefore, it does not capture the longitudinal effect of parenting on children’s outcome in both genders. Furthermore, only a correlation analysis was employed; the limitation of the analysis method is that the direction of influences among the factors cannot be determined. A longitudinal study is necessary to explore the correlations between the parenting and children’s development in different genders.
